# Discrimination, Inflammation, and Alzheimer's Disease Risk: A Study of Middle‐Aged Adults with a Family History of AD

**DOI:** 10.1002/alz70856_106930

**Published:** 2026-01-08

**Authors:** Jordan Watson, Whitney Wharton, William T. Hu, Hanfeng Huang, Patrick Gavin Kehoe, James Scott Miners, Danielle D Verble, Henrik Zetterberg, Bruno L. Hammerschlag, Lynn Marie Trotti, Karima Benameur, Brittany Butts

**Affiliations:** ^1^ Emory University, Atlanta, GA, USA; ^2^ Rutgers‐Robert Wood Johnson Medical School, New Brunswick, NJ, USA; ^3^ Rutgers Institute for Health, Health Care Policy, and Aging Research, New Brunswick, NJ, USA; ^4^ Georgetown University School of Medicine, Washington, DC, USA; ^5^ University of Bristol, Bristol, United Kingdom; ^6^ University of Bristol, Bristol, Horfield, United Kingdom; ^7^ Department of Psychiatry and Neurochemistry, Institute of Neuroscience and Physiology, University of Gothenburg, Mölndal, Sweden; ^8^ Athinoula A. Martinos Center for Biomedical Imaging, Massachusetts General Hospital, Harvard Medical School, Charlestown, MA, USA; ^9^ Harvard Medical School, Boston, MA, USA; ^10^ Massachusetts General Hospital, Boston, MA, USA; ^11^ MassGeneral Institute for Neurodegenerative Diseases, Charlestown, MA, USA; ^12^ Massachusetts General Hospital, Harvard Medical School, Boston, MA, USA; ^13^ Emory University School of Medicine, Atlanta, GA, USA; ^14^ Emory University, Altanta, GA, USA

## Abstract

**Background:**

Discrimination is a psychosocial stressor linked to poor cardiovascular and metabolic health. Chronic exposure can drive inflammation, immune activation, and vascular dysfunction, both of which are known Alzheimer's disease (AD) risk factors. Few studies have examined how types of discrimination relate to inflammation, vascular dysfunction, and neurodegeneration. We explored how situational and lifetime discrimination relates to biological mechanisms contributing to AD risk.

**Method:**

Cognitively unimpaired middle‐aged adults with a parental history of AD were enrolled in this study (PI Wharton). We measured AD, vascular and inflammatory biomarkers in brain via cerebrospinal‐fluid (Aβ40; ICAM, sPDGFRß; TGFα, MCP‐1, MDC, IL‐9, MMP‐1). Blood samples were taken to assess cortisol, inflammation markers (IL‐9, IFNγ), and vascular function (ACE‐2). Discrimination was evaluated using a battery of validated measures including Everyday Discrimination, Chronic Work Discrimination and Harassment, Heightened Vigilance, Major Experiences of Discrimination, and Coping with Discrimination.

**Result:**

Participants were 59±7 years, 70% women, college‐educated (90%), and 30% were Black Americans. Everyday discrimination was positively associated with brain [TGFα (*p* = .043), MCP‐1 ( *p* = .010), ICAM (r=.488, *p* = .011)] and peripheral [IFNγ (*p* = .031) and IL‐9 (*p* = .014)] inflammation, and was negatively associated with sPDGFRß (*p* = .015). Higher work discrimination was correlated to central MMP1 (*p* = .019), peripheral cortisol (*p* = .027), and ACE2 (*p* = .011). Major discrimination correlated with brain [MDC (*p* = .044), TGFα (*p* = .002), MCP‐1 (r=.498, *p* = .010), MMP9 (*p* = .005), ICAM (r=.547, *p* = .004)] and peripheral [IFNγ (*p* = .014) and IL‐9 (*p* = .007)]. Heightened vigilance was associated with central TGFα (r=.616, *p* = .001), Aβ40 (*p* = .002), IL‐9 (r=.440, *p* = .046), ICAM (*p* <.001), and peripheral IL‐9 (*p* = .044). Coping was positively linked to Aβ40 (*p* = .021).

**Conclusion:**

Chronic, everyday, and major discrimination correlated with higher inflammation and immune activation, both of which are risk factors for AD. Heightened vigilance (i.e. actively avoiding potential discrimination) was associated with higher inflammatory markers and Aβ_40_, while higher coping strategies correlated to increased AD biomarkers. Further research on the impact of direct (AD biomarkers) and indirect (AD risk factors) effects via discrimination is crucial, as is the need for supportive research environments and personnel to ensure participants feel comfortable sharing such experiences.

